# Safety and efficacy of therapeutic hypothermia in neonates with mild hypoxic-ischemic encephalopathy

**DOI:** 10.1186/s12887-023-04365-8

**Published:** 2023-10-26

**Authors:** Zheng Wang, Dan Zhang, Peng Zhang, Wenhao Zhou, Liyuan Hu, Laishuan Wang, Guoqiang Cheng

**Affiliations:** 1 Department of Neonatology, Shanghai General Hospital, Shanghai Jiao Tong University School of Medicine, Shanghai, 201600 China; 2 Department of Neonatology, Children’s Hospital of Fudan University, Shanghai, 200032 China; 3 Department of Fujian Key Laboratory of Neonatal Diseases, Xiamen Key Laboratory of Neonatal Diseases, Fujian, 361000 China

**Keywords:** Neonatal, Mild, Hypoxic-ischemic encephalopathy, Therapeutic hypothermia

## Abstract

**Background:**

Though there has been an increase in the number of neonates with hypoxic-ischemic encephalopathy (HIE) treated by therapeutic hypothermia (TH) in recent years, the effect of therapeutic hypothermia on mild HIE neonates is still uncertain.

**Objectives:**

This study aims to explore the safety and efficacy of therapeutic hypothermia in neonates with mild HIE.

**Methods:**

Retrospectively collected between January 2010 to December 2022 at Children’s Hospital of Fudan University, neonates with mild HIE were divided into TH and non-TH groups. Clinical data of the mild HIE neonates and their mothers’ general information during pregnancy were collected. SPSS 23.0 was used to compare the general condition, the incidence of adverse events, and efficacy in the two groups.

**Results:**

A total of 71 neonates with mild HIE were included, including 31 in the TH group and 40 in the non-TH group. Compared with the non-TH group, the TH group had significantly lower 5-minute Apgar scores [6 (5–7) points vs. 7 (5–8) points, *p* = 0.033 ], but a higher rate of tracheal intubation at birth (68%, 21/31 vs. 40%, 16/40, *p* = 0.02), a higher rate of chest compressions > 30 s (39%, 12/31 vs. 15%, 6/40, *p* = 0.023), the later initiation enteral feeding [4 (3–4) days vs. 1 (1–2) days, *p* < 0.001], a higher usage rate of analgesic and sedative drugs (45%, 14/31 vs. 18%, 7/40, *p* = 0.011) and the longer hospital stay [12.5 (11–14) days vs. 9 (7-13.9) days, *p* = 0.003]. There was no death in 71 mild HIE neonates. TH group had lower incidence of brain injury (16%, 5/31 vs. 43%, 17/40, *p* = 0.017) and encephalopathy progression (10%, 3/31 vs. 45%, 18/40, *p* = 0.001) than the non-TH group. There was no statistical significance in the incidence of adverse events between the two groups.

**Conclusion:**

Therapeutic hypothermia can reduce the incidence of brain injury in neonates with mild HIE.

**Supplementary Information:**

The online version contains supplementary material available at 10.1186/s12887-023-04365-8.

## Background

Hypoxic-ischemic encephalopathy (HIE) remains the leading cause of death and severe disability in neonates. Therapeutic hypothermia (TH) is the clinically recognized method to improve neonatal survival rate and poor prognosis of the nervous system with moderate and severe HIE [1, 2]. Studies in recent years have found that the risk of poor prognosis is increasing in mild HIE neonates, such as behavioral problems and neurodevelopmental disorders, though mild HIE has rarely been considered a risk factor for poor neurodevelopmental outcomes before [3–5]. With the advanced development of therapeutic hypothermia, the treatment rate of neonates with mild HIE has gradually increased [6]. However, few clinical trials are designed for mild HIE treated by TH, and the effect of TH on neonates with mild HIE is still unclear. Therefore, this study aims to investigate the safety and efficacy of TH in neonates with mild HIE by retrospectively analyzing the clinical data of neonates with mild HIE collected from Children’s Hospital of Fudan University in the past 13 years.

## Methods

### Criteria for inclusion and exclusion

Neonates with mild HIE were admitted to the NICU at Children’s Hospital of Fudan University from January 1, 2010 to December 31, 2022 were included.

Those who met one of the following criteria were excluded: ① gestational age < 35 weeks and birth weight < 2000 g; ② severe congenital malformations such as complex congenital heart disease, complex nervous system malformations, abnormality of trisomy- 21 and other chromosomes; ③ the neonates survived at discharge but did not have MRI data during hospitalization.

### Diagnostic criteria of mild HIE for neonates

Mild HIE was diagnosed according to the modified Sarnat criteria [7, 8] and one of the following was identified: ① level of consciousness: excitement, vulnerability to irritability; ② autonomous activity: frequent and symmetrical; ③ autonomic nervous function (generalized sympathetic nerve) : pupil dilation, tachycardia, normal or reduced gastrointestinal peristalsis; ④ primitive reflex: weak sucking reflex, strong hugging reflex, and mild tension neck reflex; ⑤ strong olfactory response; ⑥ active tendon reflex; ⑦ neuromuscular control: normal muscle tone, normal posture or mild distal curvature. Only infants with seizure onset in the 24 h after birth and confirmed on vEEG were considered as part of the progression.

### Therapeutic hypothermia

According to TH protocol in China [9], therapeutic hypothermia was carried on the whole body of neonates with the CritiCool hypothermia instrument based on routine support for the symptomatic treatment. TH protocol did not change during study periods. A rectal temperature of 33.5 ± 0.5℃ was maintained for the neonates in TH group. Rectal probes were used for continuous monitoring of the core temperature. Cooling was started within 6 h of delivery and maintained for a period of 72 h. After the cooling phase neonates were slowly rewarmed over 10–12 h (0.5℃/h). The hour age of HIE neonates at the beginning of TH, the start and end time of TH, the core body temperature of HIE neonates during TH, and the reasons for withdrawal were recorded.

In this study, 31 of 71 neonates with mild HIE received hypothermia and 40 did not. The decision to treat these neonates with hypothermia was made by neonatologists and parents in consideration of the possibility of encephalopathy progression according to birth asphyxia history, arterial blood gas analysis, clinical manifestations, and vEEG results.

### Clinical data

#### Pregnant mothers and neonates

For general information, gender, gestational age, birth weight, hour age at admission, and length of stay were recorded for neonates. For perinatal information, maternal age, mode of delivery, gestational diseases (including gestational diabetes, hypertension, and hypothyroidism), complications during delivery (including amniotic fluid contamination III, fetal heart variation, placental abruption, placenta previa, umbilical cord prolapse or true nosing, premature rupture of membranes, amniotic fluid embolism, uterine rupture, and fever) were recorded. Apgar scores at 1 and 5 min at birth, resuscitation in the delivery room or operating room (including positive pressure ventilation, tracheal intubation, chest compressions, and medical resuscitation), and arterial pH and BE values at or within 1 h after birth were recorded. For clinical manifestations of the nervous system, after the standardized nervous system physical examination by neonatologists with attending titles or above, neurological symptoms and signs, including consciousness, muscle tone, autonomic activity, primordial reflexes, convulsive seizures, central respiratory failure, pupil changes were recorded within 6 h, 24 h, and 72 h after birth, respectively.

#### Laboratory data

The worst laboratory results during hospitalization were recorded, including Platelet (PLT), C-reactive protein (CRP), alanine aminotransferase (ALT), aspartate aminotransferase (AST), creatinine (Cr), urea nitrogen (BUN), maximum glucose (GLU-max) and minimum glucose (GLU-min), prothrombin time (PT), partial prothrombin time (APTT), fibrinogen (FIB) and blood culture results.

#### Magnetic resonance imaging

Cranial MRI was performed on 1.5 (Siemens) or 3T (GE) scanners between 4 and 14 days of age. The images were analyzed and processed by a radiologist who did not know the study group allocation or the degree of encephalopathy of the subjects. Abnormal MRI was divided into [10, 11] (1) Normal or mildly abnormal: plain scan showed no abnormality, or T1WI cortical spot-like or cord-like high signal, with or without subdural hemorrhage and subarachnoid hemorrhage; (2) Moderate or severe abnormalities: abnormal signals in basal ganglia/thalamus, abnormal signals in the watershed, or diffuse injury. Normal/mildly or moderate/severe abnormal head MRI was considered no brain injury, and brain injury, respectively.

#### EEG

Video electroencephalogram (vEEG) was used to monitor brain function beside the bed with NicoletOne video brain function detector, and the monitoring time was not less than 2 h (including at least a complete sleep-wake cycle). Neonates in the TH group were monitored until 72–96 h after birth according to the situation. No sedatives were used in the 24 h before and during the examination. If the child with convulsion was given anticonvulsive phenobarbital, the dosage should be shown in the vEEG application form.

The results of the last video electroencephalography (vEEG) examination of the mild HIE neonates were recorded and vEEG results within 6 h of birth, 24 h, 72 h and before discharge were also recorded. All data were analyzed and reported by a full-time neonatal neuro electrophysiology specialist at the Department of Neonatology of Children’s Hospital of Fudan University without knowing the study group allocation. The evaluation criteria of vEEG background activity anomaly classification and paroxysmal anomaly wave are referred to the literature [12].

#### BAEP

Smart-Ep Brainstem auditory evoked potentials (BAEPs) were performed by the otolaryngologist at Children’s Hospital of Fudan University. The monitoring results were recorded during the hospitalization. The criteria for abnormal BAEPs were as follows: I ~ V waves indicated mild abnormalities with the latency of partial waves and the extension of peak interval both ≥ (‾*x* + 3s); only I and V waves indicated moderate abnormalities with prolonged intervals, irregular waveform irregular, and the amplitude ratio of V wave to I wave on the same side < 0.5; the unclear or absence of I-V wave waveform differentiation referred to the severe abnormalities [13].

#### FVEP

Flash visual evoked potentials (FVEPs) were performed by the ophthalmologist at Children’s Hospital of Fudan University through the V-ikingQuestIV evoked potentiometer produced by Nicolet. In order to avoid myoelectric artifacts, all subjects were recorded in a state of natural sleep and natural pupils during the hospitalization. Criteria for abnormal visual evoked potentials included one of the following: loss of main wave shape in one or both eyes, low main wave amplitude and prolonged latency, and a difference in latency between two eyes > 15 ms [13].

### Outcome

The primary outcomes were the mortality and the combined incidence of MRI brain injury in mild HIE neonates treated with TH.

The secondary outcome was the efficacy and safety evaluation of TH in neonates with mild HIE. Efficacy indicators included changes in encephalopathy severity, and abnormalities in cranial MRI, vEEG, and brainstem evoked potentials. Safety indicators were adverse reactions of various organs including hypotension requiring vasoactive drug treatment, PPHN requiring NO treatment with oxygen concentration > 0.50, coagulation dysfunction requiring blood transfusion treatment, hypoglycemia (whole blood Glu < 2.2 mmol/L), stress hyperglycemia (whole blood Glu > 8.3 mmol/L), and liver insufficiency (ALT > 100IU/L), renal insufficiency (Cr > 1.5 mg/dL), thrombocytopenia (PLT < 100 × 10^9^/L), and sepsis (positive blood culture and antibiotic use for more than 7 days or clinical sepsis).

### Statistical analysis

SPSS 25.0 statistical analysis software was used for data processing. The measurement data were expressed as mean ± standard deviation, and the measurement data in line with normal distribution were compared by t test. The measurement data of skewness distribution were expressed by median (interquartile spacing), and the comparison between groups was performed by Mann-Whitney U test. The statistical data were expressed by the number of cases (percentage), and the chi-square test was used for comparison between groups. Logistic regression was used to analyze the influencing factors of hypothermia in infants with mild HIE. *P* < 0.05 was considered statistically significant.

## Results

### Study sample

A total of 71 neonates with mild HIE were included, including 31 in the TH group and 40 in the non-TH group. Compared with the non-TH group, the TH group had significantly lower 5-minute Apgar scores [6 (5–7) points vs. 7 (5–8) points, *p* = 0.033 ], but a higher rate of tracheal intubation at birth (67.7%, 21/31 vs. 40%, 16/40, *p* = 0.02), a higher rate of chest compressions > 30 s (38.7%, 12/31 vs. 15%, 6/40, *p* = 0.023), the later initiation enteral feeding [4 (3–4) days vs. 1 (1–2) days, *p* < 0.001], a higher usage rate of analgesic and sedative drugs (45.2%, 14/31 vs. 17.5%, 7/40, *p* = 0.011) and the longer hospital stay [12.5 (11–14) days vs. 9 (7-13.9) days, *p* = 0.003] (Table 1). Multivariate Logistic regression analysis showed that hypothermia was the factor that delayed the start time of enteral nutrition (OR = 0.242, 95%*CI*: 0.121–0.483, *p < *0.001) (Additional file 1).

**Table 1:** Baseline Maternal and Infant Characteristics between the two groups

Variable	TH group *n* = 31	Non-TH group *n* = 40	*P* Value
Maternal Characteristics			
Age, y, mean ± SD	28.2 ± 4.2	28.3 ± 5.1	0.921
Complications of pregnancy^a^, n(%)	4 (12.9)	6 (15.0)	0.801
Intrapartum complications^b^, n(%)	22 (71.0)	21 (52.5)	0.114
Cesarean section, n(%)	7 (22.6)	13 (32.5)	0.357
**Infant Characteristics**			
Male, n(%)	20 (64.5)	22 (55.0)	0.418
Birth weight, g, mean ± SD	3307 ± 516.0	3227 ± 471.1	0.498
Gestational age, week, mean ± SD	39.5 ± 1.2	39.3 ± 1.5	0.500
Apgar score, median(IQR)			
1 min	3 (2–5)	4 (2.3-5)	0.733
5 min	6 (5–7)	7 (5–8)	0.033
Resuscitation at birth, n(%)			
Positive pressure ventilation > 10 min	10 (32.3)	6 (15.0)	0.084
Tracheal intubation	21 (67.7)	16 (40.0)	0.020
Chest compressions > 30 s	12 (38.7)	6 (15.0)	0.023
Adrenalin	7 (22.6)	5 (12.5)	0.261
Arterial blood gas in umbilical artery or 1 h postnatal, mean ± SD			
pH	7.06 ± 0.14 *n* = 19	7.02 ± 0.08 *n* = 6	0.490
BE, mmol/L	-18.7 ± 4.8 *n* = 19	-19.3 ± 4.6 *n* = 6	0.804
Initial time of Enteral feeding, day, median(IQR)	4 (3–4)	1 (1–2)	< 0.001
Analgesic and sedative drug use, n(%)	14 (45.2)	7 (17.5)	0.011
Age at admission, h, median(IQR)	4.0 (2.5-5.0)	3.3 (2.5–6.5)	0.995
Length of stay, day, median(IQR)	12.5 (11–14)	9 (7-13.9)	0.003

*Abbreviation*: *TH *Therapeutic hypothermia, *BE *Base excess, *SD *Standard deviation, *IQR *Interquartile range

^a^ Complications during pregnancy include gestational diabetes mellitus, hypertension, hypothyroidism

^b^ Complications during delivery include amniotic fluid contamination III, slow fetal heart, placental abruption, placenta previa, umbilical cord prolapse, premature rupture of membranes, amniotic fluid embolism, uterine rupture, and fever

### Comparison of efficacy

**Fig. 1:**
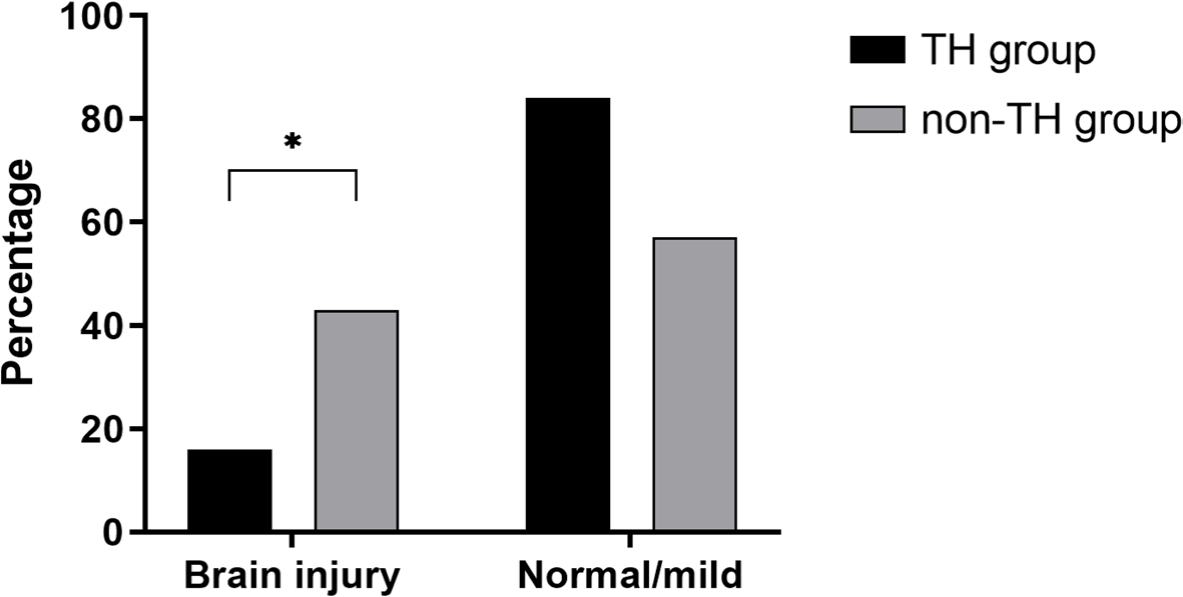
Comparison of incidence of brain injury on MRI between two groups.  Abbreviation: TH, therapeutic hypothermia; MRI, magnetic resonance imaging

There was no death in 71 neonates with mild HIE. The incidence of brain injury in the TH group (16%, 5/31) was significantly lower than that in the non-TH group (43%, 17/40) (*p* = 0.017) (Fig. 1). There was no significant difference in the incidence of abnormal vEEG, BAEP, and FVEP between the two groups.

**Fig. 2:**
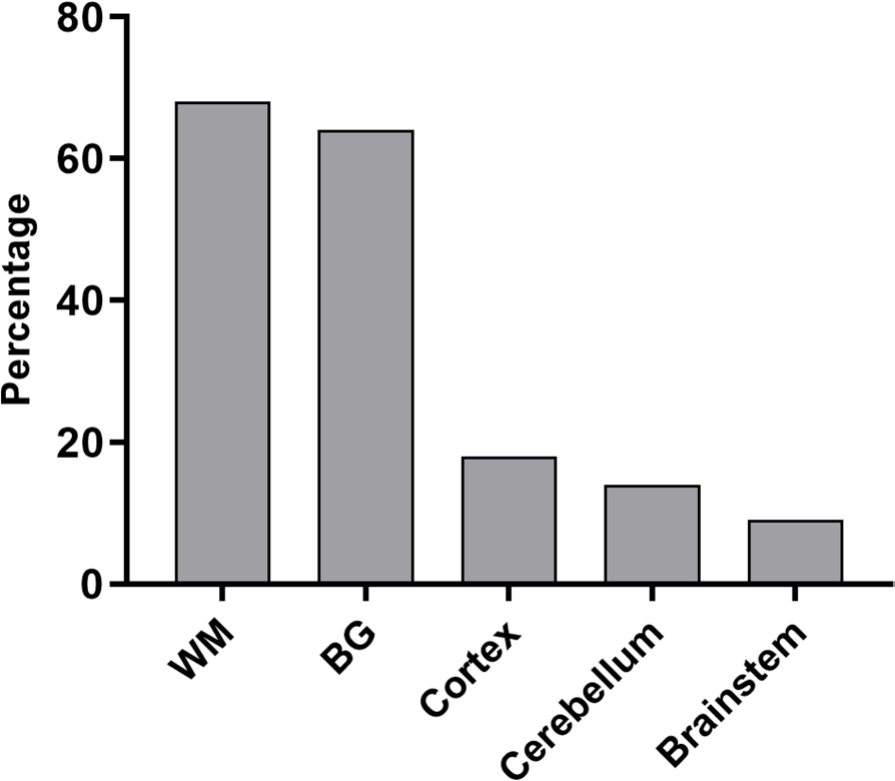
The locations of brain injure in 22 infants.  Abbreviations: WM, white matter; BG, basal ganglia

**Fig. 3:**
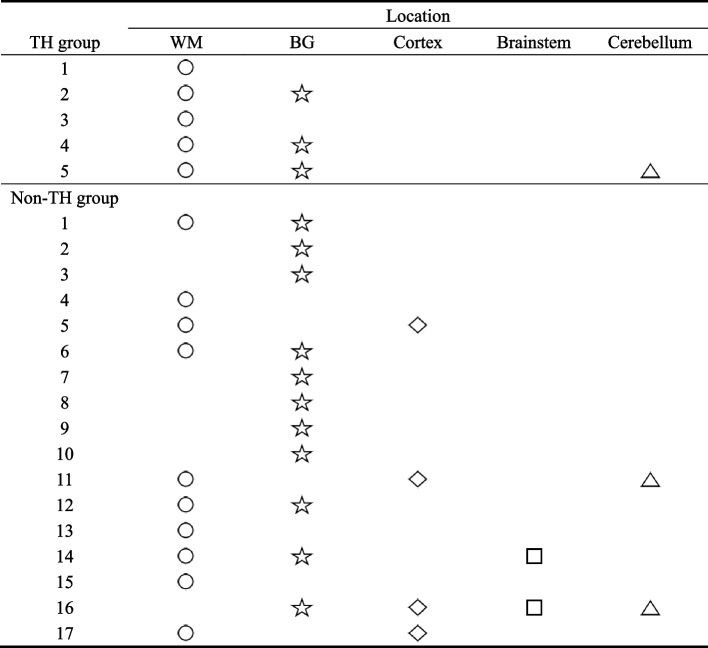
Brain injuries in TH or non-TH groups with mild encephalopathy.  Abbreviations: WM, white matter; BG, basal ganglia

The rate of white matter injury was the highest among 22 infants with brain injury, followed by basal ganglia injury (Fig. 2). In TH group, 5 infants had white matter injury, and 3 of them both had basal ganglia injury. In non-TH group, 65% (11/17) infants had basal ganglia injury, 59% (10/17) had white matter injury, and 24% (4/17) had both. 24% (4/17) of the infants in non-TH group had cortical injury, and 2 of them with white matter injury, one with white matter injury and cerebellum injury, and one with basal ganglia, brainstem and cerebellum injury (Fig. 3). There was no significant difference in the incidence of different parts of brain injury between the two groups.

### Comparison of adverse events

There was no statistical difference in the incidence of adverse events between the two groups (Table 2).

**Table 2:** Comparison of incidence of adverse events between the two groups

Variable	TH group *n* = 31	Non-TH group *n* = 40	*P* Value
Hypotension requires vasoactive drug treatment	4 (12.9)	1 (2.5)	0.218
PPHN requires NO treatment and FiO > 0.50	1 (3.2)	0 (0.0)	0.898
Blood transfusions required for coagulation dysfunction	3 (9.7)	0 (0.0)	0.157
Hypoglycemia	7 (22.6)	7 (17.5)	0.594
Stress hyperglycemia	7 (22.6)	3 (7.5)	0.142
Liver insufficiency	5 (16.1)	2 (5.0)	0.247
Renal insufficiency	2 (6.5)	0 (0.0)	0.365
Thrombocytopenia	5 (16.1)	2 (5.0)	0.247
Septicemia	5 (16.1)	4 (10.0)	0.682

*Abbreviations*: *TH *Therapeutic hypothermia, *PPHN *Persistent pulmonary hypertension, *NO N*itric oxide, *FiO* Inhaled oxygen concentration

### Progression of encephalopathy

**Fig. 4:**
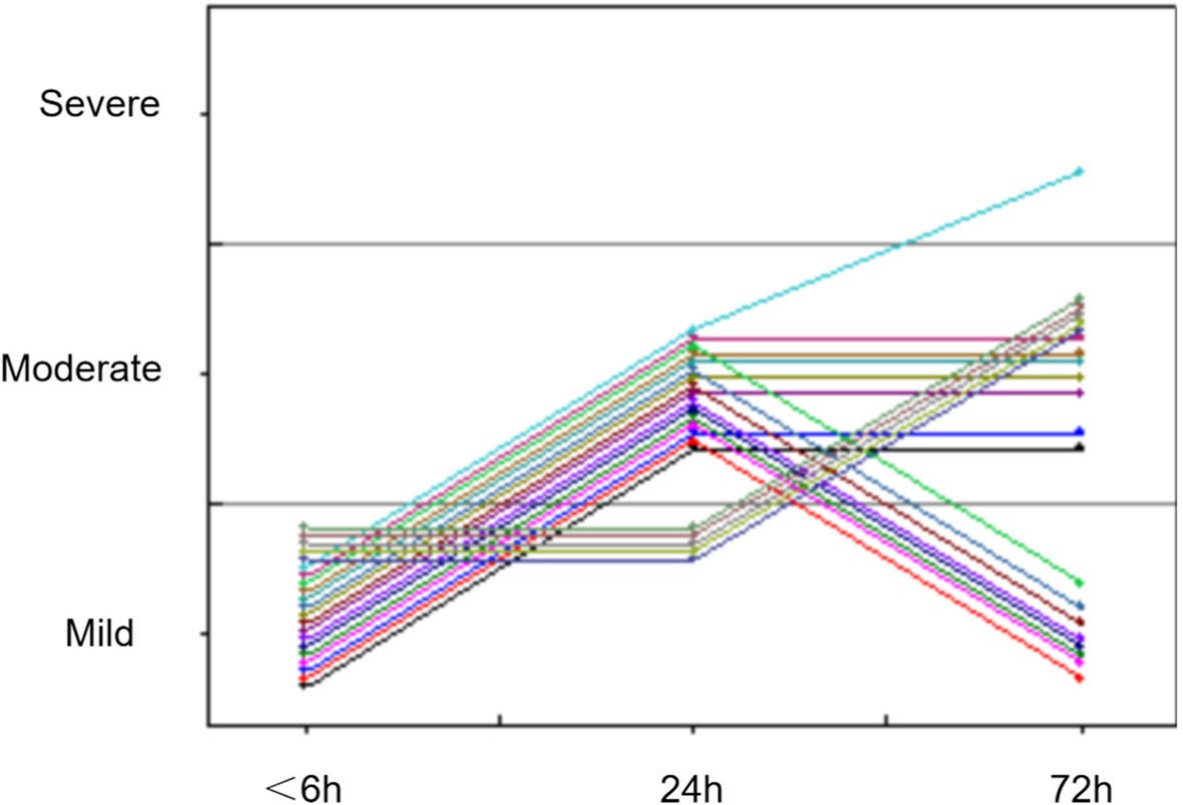
Progression of encephalopathy in 21 infants with mild HIE.  The horizontal coordinate represents the postnatal age of infants with mild HIE, and the vertical coordinate represents the severity of encephalopathy

Twenty-one infants with mild HIE had encephalopathy progression within 72 h after birth (Fig. 4). Three of them were from the TH group and 18 were from the non-TH group. The rate of encephalopathy progression in TH group (10%, 3/31) was significantly lower than that in non TH group (45%, 18/40) (*p* = 0.001).

In the TH group, all infants progressed to moderate encephalopathy with seizure onset in the 24 h after birth and confirmed on vEEG. Two infants had brain injury in MRI and one with abnormal BAEP. In the non-TH group, 9 infants progressed to moderate encephalopathy, one to severe encephalopathy, and 8 progressed to moderate encephalopathy in 24 h of life, and then improved in 72 h of life. Nine infants had brain injury in MRI and five with abnormal BAEF.

## Discussion

Mild HIE neonates were excluded from previous randomized control trials for HIE because they were generally believed to have an expected prognosis and lower risk of adverse neurodevelopmental outcomes after receiving supportive symptomatic treatment alone [3]. However, a recent meta-analysis showed that when mild HIE neonates were followed up to 3 years, the potential risk of neurological disorders accompanied by disability would increase to 24%, and the benefits of TH plus symptomatic supportive treatment outweighed the harms [14]. A prospective study by Chalak et al. found that 16% of neonates who were diagnosed with mild HIE within 6 h after birth but did not receive TH had a neurodevelopmental disorder at 18 to 22 months of age [15]. The study of Montaldo et al. also found that TH may have a neuroprotective effect on mild HIE neonates, with the increase of NAA/Cr and NAA/Cho in MRS Superior thalamus and the decrease of white matter injury on MRI [16].

However, the efficacy and safety of TH for mild HIE neonates are still unclear and need to be confirmed by RCTs in the future. Most existing literature reports are retrospective studies with small sample sizes and different results. A recent meta-analysis showed that there was insufficient evidence to prove that TH was beneficial for mild HIE neonates [17], and it may lead to more medical intervention. The results of this study showed a longer initial time of enteral feeding, higher analgesic and sedative drug use rate, and more hospital stay of mild HIE neonates receiving TH compared with non-TH mild HIE neonates, which was consistent with the findings of Gundersen [18] and Lodygensky [19] et al. However, it is worth noting that the results of this study also found that the incidence of all adverse events was higher in the TH group, although there was no statistical difference between the two groups. That indicates that it may also increase the dysfunction of other organs, although TH is safe for mild HIE neonates. Therefore, more attention should be paid to organ functions during TH for mild HIE neonates.

This study showed that the incidence of brain injury in the TH group (16%, 5/31) was significantly lower than that in the non-TH group (43%, 17/40), indicating that TH is effective in treating mild HIE neonates. A single-center retrospective study by Gagne-Loranger [20] et al. also found that the rate of brain injury in mild HIE neonates treated with TH (31%, 4/13) was lower than that in those without receiving TH (40%, 20/50). Previous studies demonstrated that infants with mild HIE have predominately white matter injury whereas infants with moderate or severe HIE are more likely to have injury to the basal ganglia and thalami [21, 22]. Our data confirm these observations. We found that 68% (15/22) mild HIE infants with brain injury had white matter injury. This is likely to reflect different underlying disease mechanisms, since white matter injury is typically associated with subacute hypoxia, whereas injury to the deep nuclei is associated with acute profound asphyxia [23]. Montaldo Paolo [16] et al. did not find any significant thalamic injury on the conventional MRI of infants with mild HIE. This is also consistent with our findings.

In the study of Gagne-Loranger et al., they found that the majority of neonates with mild HIE had an abnormal signal in the cortex regardless of whether or not they were cooled. We also found that four infants in non-TH group had cortex injury. Of note, the recent animal study suggests that TH preserves white matter when the preclinical model is subjected to mild hypoxia-ischaemia [24].

There has been only limited research thus far describing the nature of injury in mild HIE infants with progression and the effect of TH on this. Previous studies only focussed on the infants with progression who were not cooled. In our study, 21 neonates with mild HIE had further progression. Three of them from the TH group developed moderate encephalopathy, among which 2 cases had a brain injury and one with abnormal BAEP, which indicating that HIE is dynamic. Once encephalopathy progresses, there may be poor neurological prognosis. An observational cohort study by Montaldo et al. found a higher incidence of brain injury and adverse neurological outcomes in mild HIE neonates with the progression of encephalopathy [25]. However, there are few studies on TH for mild HIE neonates with encephalopathy progression. They are more vulnerable to adverse neurological outcomes. Early and reliable biomarkers to identify mild HIE neonates with the risk of encephalopathy progression also need to be further explored.

This study has several limitations. Our work is retrospective and thus prospective determinations of mild HIE for study inclusion were not made. Proper follow-up and standard neurodevelopmental assessment were lacking. It is difficult to collect and analyze the data of long-term follow-up of these mild HIE infants because of the different family economic conditions, medical conditions, convenience of medical services, follow-up scale and outcome indicators. Lastly, not all infants had vEEG, BAEP or FVEP performed. Future surveys should provide the long-term follow-up for mild HIE infants who treated with hypothermia.

## Conclusion

Based on the available evidence, mild HIE neonates may benefit from TH, but the mechanism of nerve injury in these children still needs to be investigated.Our study found that TH can reduce the incidence of brain injury in neonates with mild HIE and not increase adverse events. Neonates with mild HIE need to be included in investigations that evaluate the efficacy of emerging therapies for infants with anoxic encephalopathy.

### Supplementary Information

**Additional file 1. **Logistic regression analysis of the influencing factors of hypothermia in infants with mild HIE.

## Data Availability

The datasets used and/or analyzed during the current study are available from the corresponding author on reasonable request.
