# Supplementary material for: Safety and efficacy of therapeutic hypothermia in neonates with mild hypoxic-ischemic encephalopathy
Source: BMC Pediatr. 2023 Oct 26;23:530. doi: 10.1186/s12887-023-04365-8 (PMC10601291; doi:10.1186/s12887-023-04365-8)
Supplement: Supplementary file 1 — Additional file 1. Logistic regression analysis of the influencing factors of hypothermia in infants with mild HIE. [file 12887_2023_4365_MOESM1_ESM.docx]

**Appendix 1**

Table: Logistic regression analysis of the influencing factors of hypothermia in infants with mild HIE.

| **Variable** | **OR** | **95% CI** | ***P value*** |
| --- | --- | --- | --- |
| 5-minute Apgar scores | 1.285 | 0.943-1.750 | 0.112 |
| Tracheal intubation | 2.168 | 0.445-10.555 | 0.338 |
| Chest compressions >30 seconds | 0.488 | 0.009-27.163 | 0.726 |
| Initial time of enteral feeding | 0.242 | 0.121-0.483 | ＜0.001 |
| Analgesic and sedative drug use | 3.514 | 0.066-187.997 | 0.536 |
| Length of stay | 1.031 | 0.895-1.189 | 0.671 |

Abbreviation: HIE, hypoxic-ischemic encephalopathy; OR, odds ratio;CI, confidence interval.
